# The Impact of Controlled Ovarian Stimulation on Serum Oxidative Stress Markers in Infertile Women with Endometriosis Undergoing ICSI

**DOI:** 10.3390/antiox11061161

**Published:** 2022-06-14

**Authors:** Michele Gomes Da Broi, Elisa Melo Ferreira, Aline Zyman Andrade, Alceu Afonso Jordão, Rui Alberto Ferriani, Paula Andrea Navarro

**Affiliations:** 1 Human Reproduction Division, Department of Obstetrics and Gynecology, Ribeirão Preto Medical School, University of São Paulo, Ribeirão Preto 14048-900, SP, Brazil; mgdabroi@usp.br (M.G.D.B.); elisamferreira@yahoo.com.br (E.M.F.); amzyman@usp.br (A.Z.A.); raferria@fmrp.usp.br (R.A.F.); 2 National Institute of Hormones and Women’s Health, CNPq—Conselho Nacional de Desenvolvimento Científico e Tecnológico, Porto Alegre 90035-003, RS, Brazil; 3 Nutrition and Metabolism Laboratory, Ribeirão Preto Medical School, University of São Paulo, Ribeirão Preto 14048-900, SP, Brazil; alceu@fmrp.usp.br

**Keywords:** endometriosis, ovarian stimulation, serum, oxidative stress

## Abstract

Endometriosis-related infertility is associated with oxidative stress (OS). The present study aims to compare serum OS markers of infertile women with endometriosis and controls during the follicular phase of the natural cycle (D1), after pituitary downregulation using a GnRH agonist (D2), after controlled ovarian stimulation (COS) on the day of human chorionic gonadotropin administration (D3), and on the day of oocyte retrieval (D4). One hundred and eight serum samples (58 controls and 35 early and 18 advanced endometriosis cases) were collected at these four timepoints. OS markers were compared among the groups and timepoints using a linear regression model with mixed effects and a post-test using orthogonal contrasts. The significance was set at 5%. We observed altered OS markers in the endometriosis patients during the D1, D2, D3, and D4 timepoints compared to the controls. The evidence of systemic OS in infertile patients with endometriosis during COS suggests the mobilization of potent antioxidants in an attempt to protect the oocyte from oxidative damage, especially on the day of oocyte retrieval.

## 1. Introduction

Endometriosis is an estrogen-dependent inflammatory disease that has a prevalence of 6 to 10% among women of reproductive age, and its intriguing association with infertility remains at the center of debate in several studies [1,2,3,4,5]. The etiopathogenic mechanisms of endometriosis-related infertility are not fully understood; however, it is believed that this condition is associated with the occurrence of oxidative stress (OS), characterized by the imbalance between the production of Reactive Oxygen Species (ROS) and the adequate neutralization of such molecules by the antioxidant system [6,7,8,9]. In this regard, recent studies have found imbalanced inflammatory and oxidative markers in the peritoneal and follicular fluids of women with the disease [8,10,11,12,13,14,15,16,17,18]. Interestingly, in those patients, the oxidative stress seemed to be disseminated at the systemic level, based on the altered serum oxidative stress markers already identified [14,16,18,19,20]. Such systemic and follicular alterations have been suggested as possible mechanisms underlying the worsening of oocyte quality and, consequently, infertility in these women [15,18,21,22].

Frequently, endometriosis patients resort to Assisted Reproductive Techniques (ART) in an attempt to become pregnant [23,24]. Nevertheless, it is known that women submitted to Controlled Ovarian Stimulation (COS) to undergo ART are exposed to supraphysiological systemic concentrations of estradiol (E_2_) and progesterone (P_4_), with evidence of systemic OS due to the high doses of exogenous gonadotropins administered [25]. COS also seems to impact the follicular microenvironment by increasing ovarian metabolic activity [26,27]. As a result, ROS generation is intensified, thus favoring the occurrence of OS [28,29]. In addition, the concentrations of E_2_ and P_4_ in the follicular fluid (FF) may influence the follicular redox status, which is related to the number of retrieved oocytes in the ICSI cycles [29]. Estrogens are known to affect the redox state of the cells once they present antioxidant properties [30,31,32]. In regard to this, it is known that pituitary downregulation with analogs of GnRH preceding the administration of exogenous gonadotropins may decrease serum E_2_ concentrations [33]. Considering that it has been evidenced that patients with low serum E_2_ present increased oxidative stress in the follicular environment associated with poor-quality embryos [34], pituitary downregulation with GnRH agonists (GnRHa) might decrease the antioxidant status by reducing E_2_ levels, a fact that could intensify the OS at this point of the treatment. On the other hand, subsequent gonadotropin administration may lead to elevated E_2_ levels by stimulating multi-follicular growth [26], which could be beneficial to handle in the pro-oxidant environment. In this sense, it was observed that an increase in serum antioxidant and pro-inflammatory cytokine levels coinciding with the peak of E_2_ after COS could favor embryonic implantation and the occurrence of pregnancy [35].

However, data regarding the impact of endometriosis on ART outcomes are still conflicting. Contradictory findings suggest lower rates of fertilization, implantation, and/or live births in patients with endometriosis undergoing ART [36,37,38], as well as worse ART outcomes according to the severity of the disease [36,39]. Conversely, other studies do not suggest differences in ART outcomes among women with or without endometriosis, including their different stages, pointing to a greater impairment of outcomes due to poor ovarian reserves, secondary to maternal age or the presence of endometriomas and/or their excision [40,41,42]. In view of the low or moderate quality of evidence in those studies and keeping in mind that the data on cumulative rates remain unknown, the impact of endometriosis on ART outcomes could so far be considered to be uncertain.

Therefore, considering that endometriosis is a condition related to oxidative stress, it is unclear whether the oxidative balance of women with this disease could be modified by pituitary downregulation with GnRHa and exogenous gonadotropin administration during COS for ART, which could impact oocyte and embryo quality and the ART outcomes. However, to date, no studies have evaluated the systemic oxidative balance after a pituitary blockage with GnRHa or during COS for ICSI cycles in women with endometriosis.

Several molecules have been investigated in an attempt to understand the events related to OS. Didactically, we can consider it to be a process that occurs in three distinct phases, the first being marked by an increase in the production of reactive species, which can be analyzed using the FOX_1_ method; the second, marked by the mobilization of the antioxidant machinery as a whole, measured by the total antioxidant capacity (TAC) and enzymatic antioxidants, such as superoxide dismutase (SOD), and non-enzymatic antioxidants, including reduced glutathione (GSH) and Vitamin E (VitE), which neutralize oxidative damage in the main targets of ROS; and the third phase, characterized by the occurrence of oxidative damage to proteins, lipids, and DNA, which can be evaluated by the analysis of advanced oxidation protein products (AOPP), malondialdehyde (MDA), and 8 hydroxy-2′deoxy-guanosine (8OHdG), respectively [43].

Thus, the main objective of this study is to evaluate the presence of systemic OS in the early follicular phase of the natural cycle prior to the beginning of COS for ICSI (D1), after pituitary downregulation with GnRHa (D2), after hCG administration (D3), and on the day of oocyte retrieval (D4), comparing infertile women with endometriosis (regardless of disease staging—E, at early stages—EI/II, and at advanced stages—EIII/IV) to controls through the simultaneous analysis of the eight OS markers mentioned. As a secondary objective, this study aimed to assess the potential effects of COS on OS occurrence within the same group, by comparing the levels of the eight markers at the four COS timepoints.

## 2. Materials and Methods

### 2.1. Study Design

This prospective case-control study was conducted in the Human Reproduction Sector of the Department of Obstetrics and Gynecology of the Ribeirão Preto Medical School (FMRP-USP) and in the Laboratory of Nutrition and Metabolism of the Department of Internal Medicine, FMRP-USP. The study was approved by the Research Ethics Committee of the FMRP-USP University Hospital. All patients who fulfilled the inclusion criteria and agreed to participate in the study provided written informed consent.

### 2.2. Setting and Duration

All patients who underwent medical consultation in the Assisted Reproduction Program of the FMRP-USP University Hospital from October 2009 to October 2010 were evaluated. Those considered eligible were interviewed, and each patient who consented to participate donated a blood sample. All participants were followed up until the ART results were obtained.

### 2.3. Participants—Eligibility Criteria

In the endometriosis group, we included patients with infertility exclusively associated with endometriosis, diagnosed and classified by videolaparoscopy according to the criteria of the American Society for Reproductive Medicine (1997) [44]. All women had undergone laparoscopic surgery involving the destruction or removal of all visible endometriotic implants and the lysis of adhesions at least one year before the present study. In the control group, we included patients with infertility due to male and/or tubal factors. All control patients underwent diagnostic videolaparoscopy for the investigation of marital infertility and to rule out endometriosis and other pelvic diseases, such as active pelvic inflammatory disease.

The exclusion criteria for both groups were ages > 38 years, body mass index (BMI) ≥ 30 kg/m², serum follicle-stimulating hormone (FSH) concentrations > 10 mIU/mL on the third day of the natural menstrual cycle, the presence of polycystic ovary syndrome and other etiologies of chronic anovulation, hydrosalpinx, and chronic diseases such as diabetes mellitus or other endocrinopathies, cardiovascular disease, dyslipidemia, systemic lupus erythematosus, and other rheumatologic diseases, HIV infection, any active infection, smoking habit, and the use of hormonal medication and hormonal and non-hormonal anti-inflammatory drugs and vitamin supplements during the six months preceding the beginning of the study.

### 2.4. Assisted Reproduction Methodology

#### 2.4.1. Stimulation Protocol

Menstruation was programmed to synchronize the beginning of the COS cycle. This procedure consists of the daily administration of combined oral contraceptives during the menstrual period of the preceding cycle, up to five days before the day scheduled for the beginning of ovarian stimulation. The administration of contraceptives was discontinued, such that the beginning of menstrual bleeding would coincide with the performance of a basal transvaginal ultrasound (TVUS) to assess the endometrial pattern and rule out the presence of ovarian cysts that might interfere with the response to exogenous gonadotropins and the ultrasound monitoring of follicular growth.

Pituitary blockage with an GnRHa started 10 days before the basal TVUS (long protocol), during the evening, by the daily subcutaneous administration of 0.5 mg/day (10 IU) of leuprolide acetate (Lupron^®^, Abbott, Sao Paulo-SP, Brazil; Reliser^®^, Serono, Sao Paulo-SP, Brazil) throughout the COS period until the day of human chorionic gonadotrophin (hCG) (Ovidrel^®^, Serono, Sao Paulo-SP, Brazil) administration.

Controlled ovarian hyperstimulation was initiated five days after discontinuing the oral contraceptive pills. Each patient received 150 to 300 IU/day of recombinant FSH (FSHr) (Gonal-F^®^, Serono, Sao Paulo-SP, Brazil; Puregon^®^, Organon, Sao Paulo-SP, Brazil) subcutaneously on the first six days of induction. As of the seventh day, the dose was adjusted according to follicular growth, and endometrial thickness was monitored daily or on alternate days by TVUS.

Recombinant hCG (250 µg, Ovidrel^®^, Serono, Sao Paulo-SP, Brazil) was administered at 10:00 p.m. in the presence of at least two follicles, each measuring 18 mm in mean diameter. Oocytes were retrieved 34 to 36 h after the administration of recombinant hCG.

#### 2.4.2. Oocyte Retrieval and Denudation

Oocyte retrieval was conducted under general intravenous anesthesia with propofol (Diprivan^®^, AstraZeneca, Cotia, SP, Brazil) and fentanyl (Fentanil^®^, Janssen-Cilag, Sao Paulo, SP, Brazil). Follicles were aspirated via the endovaginal route guided by a transvaginal ultrasound transducer using a standard single-lumen needle (CDD Laboratory, Paris, France), with a constant artificial aspiration pressure of 100 mmHg provided by an electronically controlled suction pump (Craft^®^ Suction Pump, Rocket Medical, UK). Aspirated follicles were pooled in test tubes previously heated to 37 °C containing 1.0 mL of HTF-HEPES culture medium.

The aspirated material was transferred onto 10 cm diameter Petri dishes, previously heated to 37 °C on a thermal plate, containing no culture medium. After identification, the cumulus–oocyte complexes (COC) were carefully washed with HTF-HEPES culture medium (HTF, Irvine Scientific) to remove blood and debris and pooled on NUNC plates (Nunclon 4-well Multidishes, Delta SI) filled with HTF culture medium. The plates were covered with mineral oil and incubated in an oven in a 5% CO_2_ gas mixture at 37 °C and 95% humidity for 3 to 4 h. The COCs were placed into 25-µL hyaluronidase microdrops (H4272 type IV-S, SigmaAldrich, Saint Louis, MO, USA) at a concentration of 80 IU/mL in HTF/HEPES (Irvine Scientific, Santa Ana, CA, USA) for a maximum of 30 s and washed 2 to 3 times with a modified HTF medium (HTF/HEPES, Irvine Scientific, Santa Ana, CA, USA) supplemented with 10% Synthetic Serum Substitute (SSS). Cell remnants were removed using a stripper pipette (130-µm denuding pipette, Cook, Melbourne, Australia). After oocyte denudation (removal of the cumulus oophorus), the oocyte maturity was evaluated by visualization under an inverted stereomicroscope. Germinal vesicle or metaphase I stages were classified as immature oocytes and discarded, while mature oocytes (presence of an extruded polar body) were injected as described below.

#### 2.4.3. ICSI and Fertilization, Implantation, Clinical Pregnancy, and Live Birth Rates

Mature oocytes, characterized by polar body (PB) extrusion, were subjected to ICSI 3 to 4 h after retrieval. After injection, they were cultured in separate drops. Fertilization, characterized by the presence of two pronuclei and two PBs, was evaluated approximately 16 to 18 h after ICSI. The fertilization rate was calculated as the number of fertilized oocytes divided by the number of injected oocytes × 100. Embryo quality was assessed approximately 43 to 45 h after ICSI (second day of embryo development—D2) based on the number and symmetry of blastomeres, percent fragmentation, and the presence or absence of multinucleation. When embryo transfer was not performed on D2, embryo quality was analyzed again approximately 67 to 69 h after ICSI (D3).

D2 and D3 embryos were considered to be of good quality if they presented four and eight symmetrical blastomeres, respectively, without fragmentation or multinucleation [45].

Embryos were transferred on D2 or D3 depending on individual characteristics. The rate of clinical pregnancy per transfer cycle was calculated by dividing the number of patients with embryos exhibiting a heartbeat on TVUS 4 to 5 weeks after embryo transfer by the number of cycles with embryo transfer ×100. Meanwhile, the live birth rate per embryo transfer cycle was established by dividing the number of cycles with live births by the number of cycles with embryo transfer ×100.

The analyzed patient characteristics included age, weight, height, body mass index (BMI), basal FSH value on the third day of the natural cycle, the total quantity of FSH used for ovarian stimulation, the number of days of ovarian stimulation, endometrial thickness on the day of hCG administration, the total number of retrieved and mature oocytes (with extrusion of the first PB), and the total number of good-quality embryos and formed embryos.

#### 2.4.4. Sample Collection and Processing

Aliquots (5.0 mL) of venous blood were collected in sterile vacuum tubes containing ethylenediamine tetra-acetic acid (EDTA) during the follicular phase of the natural menstrual cycle before the beginning of COS for ICSI (D1), 10 days after pituitary suppression using the GnRH agonist (on the day of basal US before the beginning of COS for ICSI, D2), on the day of hCG administration (D3), and on the day of oocyte recovery (D4). The samples were centrifuged at 3000 rpm for 10 min, and the serum was divided into four aliquots and stored at −80°C for a maximum period of one year until all OS markers were determined.

The stored blood samples were sent to the Laboratory of Nutrition of FMRP-USP, where the OS markers were determined. All determinations were performed in duplicate at the same time by the same technician, who had no access to the clinical data of the patients, nor the groups to which they belonged.

#### 2.4.5. Methodology

1. Total hydroperoxides (FOX_1_)
  As previously described by our group and other researchers [18,21,46], the FOX_1_ method was applied to determine the total peroxide concentration based on the conversion of Fe^+2^ (ferrous ions) into Fe^+3^ (ferric ions) by peroxides in the analyzed samples. The results were represented as µmol/g of protein.
2. SOD
  The modified protocol from the *Labtest Diagnóstica* S.A fructosamine kit was used for SOD determination, as previously detailed by our group [18,21].
3. VitE
  The vitamin E (α-tocopherol) concentration was determined as described previously [18,21,47]. The results were expressed as μmol/L.
4. GSH—total concentration of thiols and sulfhydryl groups
  Serum GSH was determined as described previously [18,21,48], in which thiol groups reacted with dithionitrobenzoic acid (DTNB) to form a deeply colored anion with a maximum peak at 412 nm (e412 = 13,600 M^−1^cm^−1^). The concentration of sulfhydryl groups was calculated using GSH, and the results were reported as nmol/g of protein.
5. TAC
  The method based on 2,2-azinobis-3-ethylbenzothiazoline-6-sulfonate (ABTS) [49,50], previously used by our group [18,21], was applied for TAC determination. The results were reported as mEq Trolox/L.
6. AOPP
  Protein oxidation products were measured as described previously [18,21,51]. The results were expressed as µmol/L.
7. MDA
  MDA was measured based on the previously described method using TCA-TBA-HCL (15% trichloroacetic acid, 0.375% thiobarbituric acid, and 0.25 N hydrochloric acid) [18,21]. The results were represented as nmol MDA/g of protein.
8. 8OHdG
  The 8OHdG was measured by ELISA (Stressgen^®^ DNA Damage ELISA Kit, Ann Arbor, MI, USA) according to the manufacturer’s instructions, in which the 8OhdG concentration was expressed as ng/mL [18,21].
9. Determination of total proteins (FOX, MDA, and GSH, reported as g of protein)
  The Total Protein Labtest^®^ kit, which uses a methodology based on the reaction between the copper ions of the biuret reagent and the peptide bonds of the protein [18,21], was used to measure the protein content in the samples.

### 2.5. Study Size

A pilot study was carried out, in which all eligible patients who consented to participate were analyzed over a recruitment period of 12 months, from October 2009 to October 2010.

### 2.6. Statistical Analysis

An exploratory data analysis was performed through measurements of central position and dispersion and box-plot graphs. The Kruskal–Wallis test and Dunn’s post-hoc test were used to compare the clinical characteristics and the ICSI outcomes among the groups. For comparisons of markers among groups, the linear regression model with mixed effects (random and fixed effects) and the post-test using orthogonal contrasts were applied. For these analyses, the PROC MEANS and PROC MIXED procedures from the SAS^®^ 9.0 statistical program were used.

A significance level of 5% was adopted (*p* < 0.05).

## 3. Results

### 3.1. Flowchart

**Figure 1:**
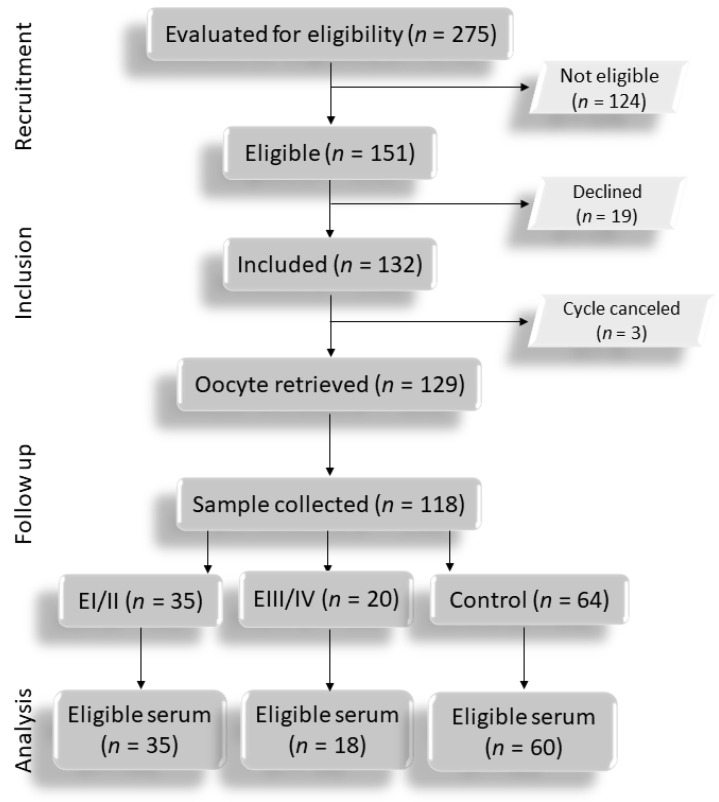
Study flowchart.

From October 2009 to October 2010, 275 patients participated in the Assisted Reproduction Program of the University Hospital of the Ribeirão Preto Medical School and underwent ovarian stimulation for ICSI. Among them, 124 were deemed ineligible. The 151 remaining patients (75 with E, 76 controls) were interviewed, and 132 (67 with E and 65 controls) provided written informed consent to participate. Blood samples were collected during the early follicular phase of the menstrual cycle that preceded the COS cycle for ICSI, after pituitary downregulation with the GnRH agonist, on the day of hCG administration, and on the day of oocyte recovery. Of these 132 patients, 129 underwent oocyte retrieval, 119 serum samples (55 with E—35 EI/II and 20 EIII/IV—and 64 controls) were donated, and 113 (53 with E—35 EI/II and 18 EIII/IV—and 60 controls) had data analyzed (Figure 1), with the remaining five considered inappropriate for OS marker determination.

### 3.2. Clinical Variables, Response to Ovarian Stimulation, and ICSI Results in Infertile Patients with Endometriosis (Regardless of Disease Staging; E), with Endometriosis I/II (EI/II), and with Endometriosis III/IV (EIII/IV) and the Controls

Significant differences regarding mean age, body mass index, baseline FSH, duration of the COS, endometrial thickness on the day of embryo transfer, the number of retrieved and metaphase II oocytes, the number of high-quality embryos, and the number of embryos formed were not observed among the evaluated groups (Table 1). Only the total dose of FSH used was significantly lower in the EI/II group (*p* = 0.03) compared to the control group and higher in the EIII/IV group compared to the control (*p* = 0.03) and EI/II (*p* = 0.03) groups. We also observed a difference in fertilization rates, which were significantly lower in the E (*p* = 0.01), EI/II (*p* = 0.02), and EII/IV (*p* = 0.02) groups compared to the control group. When comparing groups EI/II and EIII/IV with each other, it was noted that the group with the highest disease severity had lower fertilization rates (*p* = 0.02). There were no differences in implantation, clinical pregnancy, and live birth rates among the groups (Table 1).

### 3.3. Serum Oxidative Stress Markers among Infertile Patients with E, EI/II, and EIII/IV and the Controls at Different Timepoints of Controlled Ovarian Stimulation for ICSI

In the serum obtained during the early follicular phase of the cycle (D1), we observed higher concentrations of GSH in the EI/II group compared to the control group (*p* = 0.03) and the EIII/IV group (*p* = 0.01). Moreover, lower levels of TAC and 8OHdG were observed in groups E, EI/II, and EII/III (TAC: *p* < 0.01, *p* = 0.03, *p* = 0.02, respectively; 8OHdG: *p* < 0.01, *p* < 0.01, *p* = 0.04, respectively) when compared to the control group (Table 2).

Meanwhile, in the serum obtained after pituitary downregulation with the GnRHa (D2), we observed higher concentrations of GSH in the E group (*p* < 0.01) compared to the controls and a tendency toward higher levels in the EIII/IV group when compared to the control group (*p* = 0.05). Lower levels of TAC were found in the E (*p* < 0.01) and EIII/IV (*p* < 0.01) groups when compared to the controls and in the EIII/IV group compared to the EI/II group (*p* = 0.04, Table 2).

In the serum obtained on the day of hCG administration (D3), higher concentrations of GSH were observed in the E and EIII/IV groups (*p* < 0.01 and *p* = 0.01, respectively), as well as a tendency toward higher levels in the EI/II group (*p* = 0.05) when compared to the control group. In addition, we found lower levels of TAC in the E (*p* < 0.01) and EIII/IV (*p* = 0.01) groups compared to the control group. A lower concentration of 8OHdG was also noted in the E (*p* = 0.04) and EI/II (*p* = 0.01) groups when compared to the controls (Table 2).

The most significant changes in serum concentrations of the OS markers were observed on the day of oocyte retrieval (D4). Higher concentrations of FOX_1_ were found in the EIII/IV group (*p* = 0.02), and an increasing tendency was noted in the E group (*p* = 0.05) when compared to the controls. Higher levels of SOD were observed in the E (*p* < 0.01) and EIII/IV (*p* < 0.01) groups compared to the controls and in the EIII/IV group compared to the EI/II group (*p* < 0.01). An upward trend was also noted in the concentration of GSH in the E group (*p* = 0.05) compared to the control group. Lower levels of TAC were found in the three endometriosis groups (E: *p* < 0.01; EI/II: *p* = 0.02, and EIII/IV: *p* < 0.01) when compared to the controls. In addition, increased concentrations of AOPP were observed in the EI/II group (*p* = 0.04) compared to the control group (Table 2).

No statistically significant differences were found among groups regarding MDA and VitE levels.

### 3.4. Serum Oxidative Stress Markers among the Different Timepoints of Controlled Ovarian Stimulation for ICSI within the Same Group of Infertile Patients with E, EI/II, and EIII/IV and the Controls

In the control group, we found higher levels of FOX_1_ in the serum obtained during the early follicular phase of the cycle (D1) compared to the serum obtained after the pituitary blockage with the GnRH analog (D2) (*p* = 0.01), on the day of the hCG administration (D3) (*p* = 0.01), and on the day of oocyte retrieval (D4) (*p* < 0.01). Lower concentrations of SOD were observed in D3 compared to D1 (*p* < 0.01), D2 (*p* = 0.03), and D4 (*p* = 0.04). A trend toward lower levels of TAC was observed in D3 compared to D1 (*p* = 0.05). Higher concentrations of AOPP were found in D2 compared to D1 (*p* < 0.01) and D4 (*p* = 0.04). We noted a trend toward higher levels of MDA in the serum obtained in D2 compared to that obtained in D1 (*p* = 0.05) and significantly higher levels of this marker in D2 compared to that obtained in D4 (*p* = 0.03, Table 3).

In the E group, higher levels of FOX_1_ were found in D1 compared to D2 (*p* < 0.01), as well as to D3 (*p* < 0.01). Higher concentrations of SOD were observed in D4 compared to D1 (*p* < 0.01), D2 (*p* < 0.01), and D3 (*p* < 0.01). We noted a trend toward lower levels of GSH in D1 compared to D2 (*p* = 0.05) and significantly lower concentrations of GSH in D1 compared to D3 (*p* < 0.01) and D4 (*p* < 0.01). TAC levels were higher in D1 compared to D3 (*p* = 0.02) and D4 (*p* = 0.03), and there was a trend toward higher levels of this marker in D2 compared to D3 (*p* = 0.05). Higher concentrations of AOPP were observed in D2 compared to D1 (*p* < 0.01, Table 3).

As for the EI/II group, a trend toward higher levels of FOX_1_ was observed in D1 compared to D2 (*p* = 0.05), as well as significantly higher FOX_1_ levels in the serum obtained in D1 compared to D3 (*p* = 0.01). Higher concentrations of SOD were observed in D4 compared to D2 (*p* = 0.03). We also found higher levels of GSH in D4 compared to D1 (*p* = 0.02). TAC levels were higher in D2 compared to D3 (*p* = 0.02) and D4 (*p* = 0.01). Lower concentrations of AOPP were observed in D1 compared to D2 (*p* < 0.01) and D4 (*p* < 0.01, Table 3).

In the EIII/IV group, we observed higher levels of FOX_1_ in D1 than in D2 (*p* = 0.01). Higher levels of SOD were observed in D4 compared to D1 (*p* < 0.01), D2 (*p* < 0.01), and D3 (*p* < 0.01). We also found lower levels of GSH in D1 compared to D3 (*p* < 0.01) and D4 (*p* < 0.01). TAC levels were also lower in D4 compared to D1 (*p* = 0.03, Table 3).

There were no significant differences in the levels of VitE and 8OHdG among the timepoints in any of the studied groups.

## 4. Discussion

OS has been identified as one of the leading factors involved in the etiopathogenesis of infertility associated with endometriosis [6,7,8]. Interestingly, endometriosis patients are frequently submitted to ART in an attempt to become pregnant [23,24], although there is no consensus regarding the disease’s impact on in vitro fertilization (IVF) outcomes [36,37,38,40,41,42]. Nevertheless, it is known that pituitary downregulation with GnRHa and ovarian stimulation with exogenous gonadotropins may influence ovarian metabolic activity, as well as E_2_ levels, and modify the redox status of the follicular and systemic environments in infertile women submitted to ART [25,26,27,28,29,33]. In this context, it is unclear whether the serum oxidative status of women with endometriosis undergoing ART could be impacted by COS, a fact that could affect their outcomes. However, to date, there are no studies analyzing the pro- and antioxidant serum profiles at different timepoints of COS for ICSI within the same group of infertile women with endometriosis and among its different stages. Knowing the oxidant and antioxidant profiles of these patients would allow us to suggest therapeutic approaches to be followed prior to or during COS in order to improve their ART outcomes. Thus, in the present study, we compared the serum levels of eight pro-oxidant (FOX_1_, MDA, AOPP, and 8OHdG) and antioxidant (GSH, Vitamin E, SOD, and TAC) OS markers from serum samples obtained in the follicular phase of the cycle prior to COS (D1), after 10 days of pituitary downregulation with a GnRHa (D2), on the day of hCG administration (D3), and on the day of oocyte retrieval (D4) among controls and women with E, EI/II, and EIII/V. In addition, we compared the serum levels of the eight markers among the different COS timepoints mentioned within the same groups of patients.

Based on the results obtained by comparing the endometriosis and control groups, we found evidence of the occurrence of systemic OS in infertile women with endometriosis, even in the follicular phase of the natural cycle preceding COS (D1). Higher GSH concentrations were observed in the serum obtained in D1 in the EI/II group compared to the control and EIII/IV groups. The higher GSH concentrations in the EI/II group than in the control group suggest a greater mobilization of this important antioxidant in a probable attempt to prevent systemic OS. The higher levels of GSH found in the EI/II group compared to the EIII/IV group corroborate the findings reported by Andrade et al. (2010) and suggest a greater mobilization of the antioxidant system in the group with early stages of the disease to prevent oxidative damage at the systemic level [19]. In inflammatory processes, an intense production of hydroperoxides and organic peroxides occurs, possibly inducing the mobilization of GSH due to its fundamental role in the neutralization of these compounds [52]. The reduced form of glutathione is frequently utilized by the enzymes of the glutathione family as a H^+^ donor for the degradation of peroxides, and GSH depletion results in DNA damage and increased hydrogen peroxide levels [9]. An inversely proportional relationship between GSH and 8OHdG was demonstrated in human fibroblast cultures treated with a carcinogenic substance, with reduced GSH levels leading to increased 8OhdG levels [53]. Thus, we hypothesize that increased serum GSH concentrations favored the reduction of serum 8OHdG levels observed herein. The lower levels of 8OHdG found in the EIII/IV group, however, contrast with the lower levels of GSH observed, which raises the hypothesis that we may have accessed a moment of intense consumption of GSH in this group, since the levels of this antioxidant in the other three timepoints studied were always greater than those observed in the other groups when analyzing the data altogether. In addition, we noted a reduction in TAC in the serum of women with endometriosis (all three groups), suggesting a decreased overall antioxidant capacity, indicating the occurrence of systemic OS, which may participate in the reduced natural fertility of these women.

Pituitary downregulation with GnRH agonists (D2), widely used in ART, causes the desensitization of GnRH receptors, reducing the serum availability of gonadotropins and, consequently, leading to lower production of E_2_ by follicular cells [54,55]. Since lower levels of E_2_ are related to decreased antioxidant activity and increased oxidative stress in the follicular microenvironment [34], pituitary downregulation with GnRHa could lead to intense OS at this point of the treatment. On the other hand, given that endometriosis is an estrogen-dependent disease, the decrease in E_2_ due to GnRHa treatment, reducing E_2_ availability, could contribute to the lower proliferation of endometriotic foci [4,54], decreasing the OS in the endometriosis groups. In this sense, unlike what was observed in the natural cycle, after pituitary downregulation with GnRHa, no differences in OS markers were observed in the EI/II group compared to the control group, suggesting that, in the early disease group, short pituitary downregulation with GnRHa can restore oxidative homeostasis at the systemic level. Conversely, in the advanced disease group, pituitary downregulation was not able to control systemic OS, with the maintenance of higher concentrations of GSH and lower TAC when compared to the control group being similar to what was observed in D1.

The use of gonadotropins to induce COS may lead to elevated serum levels of E_2_ [56], which are more pronounced on the trigger day (D3). Otherwise, under the effects of ovarian stimulation, some intensification of OS could be expected on the day of hCG administration, especially in the EIII/IV group, due to the increase in endometriotic foci activity in response to the higher levels of circulating E_2_. Moreover, exogenous ovarian stimulation is related to increased ovarian metabolic activity [26,27], which may intensify ROS generation, thus intensifying OS [28,29]. However, we did not find significant changes in the concentrations of the pro-oxidant markers and observed an OS pattern similar to that revealed in D1, with higher concentrations of GSH and lower 8OHdG and TAC levels in the endometriosis groups. Thus, it is possible that the absence of any detectable difference in the levels of pro-oxidant agents at this stage may reflect a potential additional protective role of E_2_ itself. Estradiol is involved in the proliferation of endometriotic lesions and, paradoxically, also exerts a powerful antioxidant effect, modulating the activity of OS-neutralizing enzymes [30,31,32]. It has been shown that E_2_ may restore the levels of glutathione peroxidase, catalase, thioredoxin 2, and peroxiredoxin 3 affected by the pro-oxidizing heavy metal Chromium, as observed in the ovaries of rats whose mothers were exposed to this agent [57]. In addition, E_2_ appears to act directly on H_2_O_2_ modulation in apoptotic and cell proliferation processes in endometrial cells [58].

It is known that serum estradiol concentrations start to decrease after hCG triggering [59]. This reduction in E_2_, associated with greater follicular metabolic activity at that moment, could explain the slightly different findings in D4 in relation to D3. Thus, on the day of oocyte retrieval, especially in EIII/IV patients, the decrease in E_2_, associated with intense follicular metabolic activity, may lead to systemic OS, evidenced by the increase in FOX_1_ and SOD and the maintenance of lower TAC levels compared to the controls. In the early endometriosis group, systemic OS was evidenced by the persistence of reduced TAC and the increase in AOPP in relation to the control group. The fact that we found higher levels of FOX_1_ in the EIII/IV group compared to the controls suggests higher ROS generation in the more severe group. The evidence of protein damage in the serum compartment would explain the higher levels of powerful antioxidants such as SOD and GSH on a day that coincides with intense follicular metabolic activity and the final events of oocyte nuclear maturation, whose progress has already shown to be altered locally by oxidative damage caused by pro-oxidant agents present in the follicular fluid [15,60] and possibly also due to systemic changes. In a previous study conducted by our group, a higher expression of the *SOD1* gene in cumulus cells collected on the day of oocyte retrieval was also observed in women with EIII/IV, suggesting greater mobilization of this antioxidant in an attempt to protect the oocyte from oxidative agents [61]. The fact that we did not find any changes in the 8OHdG levels indicates that, perhaps, such neutralization was effective in preventing potentially harmful oxidative effects on oocyte DNA. The high levels of SOD in the EIII/IV group compared to the EI/II group reinforce the idea that this enzyme is strongly associated with oxidative protection, according to the severity of the disease. Similarly, Amreen et al. (2019) reported lower superoxide dismutase levels with the increased severity of endometriosis in blood samples collected during the follicular phase of natural cycles of fertile and infertile patients, although no statistically significant differences were found [7]. However, to date, no studies have compared the levels of this marker in serum obtained on the day of oocyte retrieval among the different stages of endometriosis.

Among all the analyzed ovarian stimulation and ICSI outcomes, only the total amount of FSH used and the fertilization rates were lower in the EI/II group compared to the controls, corroborating the findings described by Opøien et al. (2012, who also observed lower rates of fertilization in this group of patients [38]. When comparing the two endometriosis subgroups, we observed a lower rate of fertilization in the group with the more severe form of the disease, without, however, affecting the live birth rates in either group. Altogether, these data suggest that the antioxidant mobilization during COS, especially on the day that coincides with the final events of oocyte nuclear maturation, could avoid oocyte damage, even in patients with severe stages of the disease.

When analyzing the possible effects of pituitary downregulation and gonadotropin stimulation for ICSI on the occurrence of OS in each group individually (intra-group analysis), we observed a higher generation level of ROS in D1 in the control group (increased serum levels of FOX_1_) compared to that found in the analyses carried out under the effects of pituitary blockage with the GnRHa (D2) and COS (D3 and D4). The fact that we found higher serum levels of FOX_1_ in D1, in a group considered to be a physiological standard, could be justified by the role of ROS in the regulatory signaling necessary for ovary function, which is crucial for oocyte health. Considering that, under physiological conditions, there is a complete balance between ROS and antioxidant defense, which may protect against oxidative damage [43], a reduction in ROS with pituitary downregulation and COS is expected and, perhaps, might impact correct cellular signaling. In the analyzed endometriosis groups, higher levels or a tendency toward higher levels of FOX_1_ were also detected in this phase compared to the other moments of COS, which would confirm this hypothesis. The fact that the TAC levels tended to be higher in D1 when compared to D3 (already under the effects of COS) in the control group suggests the mobilization of the antioxidant system to balance the ROS generated in this period. We also observed increased serum levels of AOPP and an upper trend of MDA after pituitary downregulation (D2) compared to the observed levels of these markers in the natural cycle. Since MDA and AOPP are products formed as a result of a cascade initiated from the generation of H_2_O_2_, it seems reasonable that the high levels of these markers in D2 could be a consequence of the decrease in circulating E_2_ levels after GnRHa administration, preventing its antioxidant action. The lower levels of MDA and AOPP found in D4 compared to those observed in D2 would confirm the recovery of the physiological levels of these markers in the last analyzed stage of COS, the day of oocyte retrieval. Unlike Kaya et al. (2004), who found a positive correlation between increased serum levels of estradiol and higher serum levels of MDA during ovarian stimulation (corresponding to D3 in the present study), when there is a peak in E_2_ [56], we did not detect any differences in the levels of this marker at that stage. It is important to highlight the decrease in serum SOD levels in D3 compared to the other analyzed moments (D1, D2, and D4). We ponder the occurrence of the higher consumption of this enzyme in this period, effectively neutralizing any possible oxidative processes, since we did not detect any evidence of alterations in the levels of pro-oxidant agents. On the other hand, it is possible that this decrease is related to the increased levels of E_2_ expected for that period, as demonstrated by Lutosławka et al. (2003), who reported an inhibitory effect of estradiol on SOD activity in women with normal ovulation [62]. However, although we found evidence of lipid and protein peroxidation at the end of the downregulation with GnRHa in this group, we did not detect changes in the levels of Vitamin E, the main antioxidant responsible for neutralizing these compounds [43]. We also did not observe changes in the levels of 8OHdG, a marker of DNA damage.

In the endometriosis group, the observation of lower concentrations of GSH in the early follicular phase of the natural cycle compared to D2, D3, and D4 may suggest that pituitary downregulation and COS promoted greater mobilization of this antioxidant. Moreover, considering that a decrease in E_2_ levels and, consequently, a lower antioxidant defense with the administration of GnRHa are expected, despite the occurrence of a reduction in FOX_1_ (decline in hydroperoxide production), the data show an increasing tendency in GSH and an increase in AOPPs, indicating protein oxidative damage after the previous increase in ROS, even with a suggestive antioxidant response. Likewise, COS promoted a reduction in FOX_1_, an increase in SOD and GSH, and a reduction in TAC. These alterations suggest that, even with the greater mobilization of important antioxidants, there was consumption of the antioxidant defense, probably in an attempt to prevent the OS generated by the increase in follicular metabolic activity. AOPPs are serum aggregates of OS-oxidized albumin [63], and their detection after ovarian suppression would confirm the participation of ovarian hormones in the control of protein damage. Toth et al. (2006) demonstrated that the use of a GnRH analog interfered with protein metabolism in women, although protein oxidation was not observed [64]. We highlight the fact that the serum concentrations of GSH increased throughout the cycle, reaching higher levels in the final stages, after COS, and on the day of oocyte retrieval. Enzymes of the glutathione family, such as glutathione peroxidase, are present in the preovulatory follicle and keep hydroperoxide levels low, playing fundamental roles in gametogenesis and fertilization [43]. We also emphasize that there was a pronounced increase in serum SOD levels on the day of oocyte retrieval (D4) compared to the other analyzed timepoints, suggesting a strong mobilization of potent antioxidants, possibly to protect the oocyte against ROS generated in D2 and D3 that were not completely neutralized, or even oxidative damage promoted during COS.

When analyzing the serum of patients with early stages of endometriosis (EI/II), we noted a reduction in hydroperoxides after pituitary downregulation and COS compared to the natural cycle. However, a mobilization of important antioxidants on the day of oocyte retrieval was observed, as well as the consumption of the antioxidant capacity with COS, culminating in protein oxidative damage (demonstrated by increased AOPP). Thus, similarly to the E group, early endometriosis stages were also related to OS during GnRHa administration and COS, and the antioxidant mobilization was not able to prevent oxidative damage. Regarding the advanced stages of the disease (EIII/VI), a reduction in hydroperoxides with pituitary downregulation was also observed, as was a greater mobilization of GSH after COS. Likewise, we noted an increase in SOD and a reduction in TAC on the day of oocyte retrieval. Interestingly, there was no evidence of alterations in AOPP in this group. These data suggest that, despite the evidenced OS, the antioxidant defense was efficient in preventing protein oxidative damage, with a significant mobilization of SOD on the day of oocyte aspiration. A fact that intrigued us in these groups was the marked mobilization of more specific antioxidants, such as GSH and SOD, mainly on the day of follicle aspiration, coinciding with the final stages of oocyte maturation. Younis et al. (2012) had already reported an increase in serum levels of SOD and GSH related to ovarian stimulation [35], which was confirmed in the present study. It has also been shown that the luteinizing hormone (LH), present at high levels in the preovulatory follicle, may stimulate the production of intrafollicular superoxide dismutase in rats [65], which we believe to happen also occurs at a systemic level.

This study’s main limitation was the evaluated sample size, which was small due to the extremely strict eligibility criteria, limiting the generalizability of the study. Nevertheless, it was necessary in order to minimize possible biases in sample selection, avoiding the interference of other factors associated with oxidative stress and impaired oocyte quality and increasing the study’s internal validity. In addition, a strict protocol for sample collection was adopted since the blood was obtained prior to the application of the anesthetic in order to avoid possible biases resulting from anesthetic stress and from the ovarian puncture itself, which could influence the levels of the OS markers analyzed.

## 5. Conclusions

In conclusion, when comparing the serum OS markers throughout COS in the studied groups among themselves (inter-group analysis), we found evidence of greater OS in the endometriosis groups (E, EI/II, and EIII/IV) at different timepoints, suggesting an impact of pituitary downregulation with GnRHa and ovarian stimulation on the oxidative balance of these patients, especially on the day of oocyte retrieval. When analyzing the possible effects of COS for ICSI on the occurrence of OS in each group individually (intra-group analysis), we observed an intense generation of ROS in the early follicular phase, both in the control group and the endometriosis groups. In addition, we demonstrated that pituitary downregulation and ovarian stimulation with gonadotropins generated OS and mobilized the antioxidant system. In groups EI/II and EIII/IV, the mobilization of potent antioxidants was markedly accentuated on the day of oocyte retrieval as a potential attempt to protect the oocyte from oxidative damage. The fact that we did not find differences in the ICSI outcomes among the endometriosis and control groups reinforces the hypothesis that, at the beginning of ROS generation, the total antioxidant capacity was efficient in repressing the oxidative damage promoted in the cycle prior to COS, and, under gonadotropin use, more specific antioxidants take on such protection, suggesting that the prompt neutralization of reactive species may be able to reduce the oxidative effects without compromising the oocyte quality and, consequently, gestational success.

## Figures and Tables

**Table 1:** Clinical variables, responses to ovarian stimulation, and ICSI outcomes for infertile patients without endometriosis and with endometriosis, EI/II, and EIII/IV who underwent controlled ovarian stimulation.

Variable	Control	Endometriosis (E)	Endometriosis I/II (EI/II)	Endometriosis III/IV (EIII/IV)
Age (years)	33.28 (±4.56)	32.93 (±3.27)	32.94 (±3.39)	32.68 (±3.73)
BMI (kg/m^2^)	24.12 (±3.4)	24.05 (2.9)	24.05 (±2.9)	23.84 (±4.19)
Baseline FSH	5.52 (±1.94)	5.74 (±2.3)	5.78 (±2.3)	5.48 (±2.85)
Total dose of FSH	1998.45 (±830.2) ^ab^	2044.43 (±657.39)	1820.83 (±584.21) ^ac^	2392.5 (±673.73) ^bc^
Days of stimulation	8.42 (±2.01)	8.78 (±1.74)	8.47 (±1.81)	9.39 (±1.33)
Endometrial thickness (mm)	12.63	10.73 (±1.71)	10.63 (±1.64)	10.48 (±1.94)
Number of retrieved oocytes	5.89 (±3.54)	7.36 (±5.06)	8.03 (±5.23)	7.44 (±4.97)
Number of metafase II oocytes	4.56 (±3.1)	5.35 (±3.15)	5.62 (±2.98)	5.67 (±3.51)
Fertilization rate (%)	81.08% ^abc^	72.31% ^a^	73.19% ^bd^	67.77% ^cd^
Number of high-quality embryos	1.00 (±1.19)	1.18 (±1.47)	1.38 (±1.61)	1.00 (±1.32)
Number of formed embryos	2.72 (±1.35)	2.75 (±1.44)	3.00 (±1.44)	2.53 (±1.55)
Number of cycles with fresh embryo transfer	30	31	30	17
Clinical pregnancy rate (%)	16/48 (33.33%)	19/46 (41.3%)	11/33 (39.29%)	6/33 (42.84%)
Live birth rate (%)	13/48 (27.08%)	12/47 (25.53%)	7/29 (24.14%)	3/29 (21.43%)

Note: BMI: Body Mass Index. Data presented as mean ± standard deviation or rate (percentage). The same superscript letters in a line indicate a significant difference.

**Table 2:** Oxidative stress (OS) markers in serum samples obtained during the early follicular phase of the natural cycle prior to the beginning of controlled ovarian stimulation for ICSI (D1), after pituitary downregulation with GnRHa (D2), after hCG administration (D3), and on the day of oocyte retrieval (D4), comparing infertile women with endometriosis (regardless of disease staging—E), with early endometriosis (EI/II), and with advanced endometriosis (EIII/IV) and infertile controls (C).

Time-Point	OS Marker	C (*n* = 60)	E (*n* = 53)	EI/II (*n* = 35)	EIII/IV (*n* = 18)
**D1**	FOX_1_	8.38 (7.85–8.92)	8.81 (8.32–9.30)	8.77 (8.18–9.36)	9.23 (8.00–10.46)
	SOD	589.87 (516.44–663.30)	554.82 (511.10–598.54)	534.63 (488.73–580.53)	580.07 (479.07–681.07)
	VitE	22.06 (20.50–23.63)	22.3 (20.41–24.19)	21.56 (19.28–23.84)	22.16 (17.96–26.36)
	GSH	181.91 (167.15–196.67) ^a^	191.38 (175.29–207.46)	189.48 (170.37–208.60) ^ab^	179.26 (142.81–215.70) ^b^
	TAC	0.49 (0.45–0.53) ^abc^	0.40 (0.34–0.45) ^a^	0.40 (0.33–0.46) ^b^	0.39 (0.30–0.48) ^c^
	AOPP	103.45 (87.59–119.32)	113.84 (100.12–127.55)	104.96 (89.29–120.62)	119.97 (92.81–147.14)
	MDA	18.96 (15.85–22.07)	19.67 (17.17–22.17)	19.81 (16.18–23.45)	18.99 (14.14–23.84)
	8OHdG	21.27 (19.06–23.49) ^abc^	16.92 (15.12–18.73) ^a^	15.81 (14.18–17.44) ^b^	16.92 (11.67–22.18) ^c^
**D2**	FOX_1_	7.76 (7.30–8.22)	7.82 (7.45–8.19)	8.09 (7.56–8.63)	7.45 (6.70–8.19)
	SOD	560.0 (499.45–620.55)	520.69 (485.0–556.39)	507.43 (454.01–560.85)	537.15 (467.85–606.45)
	VitE	21.4 (19.62–23.18)	22.55 (20.96–24.14)	22.83 (20.47–25.19)	22.74 (19.19–26.29)
	GSH	181.26 (168.74–193.78) ^a^*	211.13 (199.29–222.96) ^a^	201.78 (183.82–219.74)	216.25 (198.29–234.20) *
	TAC	0.50 (0.46–0.53) ^ab^	0.39 (0.33–0.46) ^a^	0.44 (0.35–0.53) ^c^	0.36 (0.23–0.49) ^bc^
	AOPP	136.05 (115.39–156.70)	146.98 (126.49–167.46)	146.19 (117.38–177.01)	152.00 (110.92–193.08)
	MDA	20.66 (16.95–24.38)	19.26 (16.01–22.50)	19.29 (14.77–23.82)	16.63 (10.27–23.00)
	8OHdG	20.23 (18.25–22.20)	19.08 (16.66–21.49)	18.53 (15.59–21.48)	20.19 (14.28–26.09)
**D3**	FOX_1_	7.71 (7.12–8.29)	7.69 (7.32–8.06)	7.72 (7.16–8.27)	7.89 (7.11–8.67)
	SOD	481.48 (431.22–531.75)	499.93 (433.49–566.37)	526.34 (453.26–599.42)	485.83 (305.34–666.32)
	VitE	22.22 (19.93–24.51)	23.05 (20.92–25.17)	21.94 (19.23–24.66)	23.98 (19.0–28.97)
	GSH	188.28 (176.88–199.69) ^ab^*	222.13 (212.58–231.68) ^a^	214.18 (200.72–227.64) *	235.45 (217.51–253.38) ^b^
	TAC	0.43 (0.40–0.47) ^ab^	0.33 (0.27–0.39) ^a^	0.35 (0.27–0.42)	0.31 (0.19–0.43) ^b^
	AOPP	124.68 (103-57–145.79)	132.84 (112.28–153.40)	135.23 (104.30–166.15)	126.76 (89.23–164.30)
	MDA	18.94 (15.37–22.51)	18.81 (15.73–21.88)	19.67 (15.68–23.65)	16.43 (9.69–23.18)
	8OHdG	21.45 (19.19–23.70) ^ab^	18.37 (16.21–20.53) ^a^	17.06 (14.33–19.79) ^b^	18.80 (14.98–22.62)
**D4**	FOX_1_	7.66 (7.15–8.17) ^a^*	8.22 (7.72–8.72) *	8.38 (7.61–9.14)	8.36 (7.61–9.12) ^a^
	SOD	564.66 (511.83–617.49) ^ab^	666.69 (576.13–757.25) ^a^	619.89 (492.77–747.01) ^c^	812.78 (651.02–974.55) ^bc^
	VitE	20.80 (18.62–22.97)	23.72 (21.52–25.92)	23.78 (21.17–26.39)	23.77 (18.66–28.89)
	GSH	196.83 (184.12–209.55) *	220.51 (207.54–233.49) *	219.52 (200.91–238.12)	231.82 (210.38–253.27)
	TAC	0.45 (0.41–0.50) ^abc^	0.34 (0.29–0.39) ^a^	0.34 (0.27–0.42) ^b^	0.29 (0.20–0.37) ^c^
	AOPP	115.26 (96.23–134.29) ^a^	133.83 (112.16–155.51)	149.88 (117.60–182.17) ^a^	121.47 (84.67–158.28)
	MDA	18.82 (15.5–22.14)	19.45 (15.64–23.27)	18.96 (13.51–24.41)	18.0 (11.62–24–37)
	8OHdG	19.38 (17.35–21.40)	16.57 (15.30–17.85)	16.05 (14.58–17.52)	17.36 (14.28–10.44)

Note: Data presented as mean (95% Confidence Interval) and compared among groups using a linear regression mixed-effects model and an orthogonal contrast post-test. Significance was defined as *p*-value < 0.05. FOX_1_ (total hydroperoxides): μmol/g protein; MDA (malondialdehyde): nmol/g protein; AOPP (advanced oxidation protein products): μmol/L; 8OHdG (8-hydroxy-2′-deoxyguanosine): ng/mL; GSH (reduced glutathione): nmol/g protein, VitE (vitamin E): μmol/L; SOD (superoxide dismutase): U/mL; TAC (total antioxidant capacity): mEq Trolox/L. The same superscript letters in a line indicate a significant difference. *: *p* = 0.05.

**Table 3:** Oxidative stress (OS) markers in serum samples obtained during the early follicular phase of the natural cycle prior to the beginning of controlled ovarian stimulation for ICSI (D1), after pituitary downregulation with the GnRHa (D2), after hCG administration (D3), and on the day of oocyte retrieval (D4), comparing the four timepoints within each group: infertile women with endometriosis (regardless of disease staging—E), with early endometriosis (EI/II), and with advanced endometriosis (EIII/IV), and infertile controls (C).

Group	OS Marker	D1	D2	D3	D4
**C** **(*n* = 60)**	FOX_1_	8.38 ^abc^	7.76 ^a^	7.71 ^b^	7.66 ^c^
	SOD	589.87 ^a^	560.00 ^b^	481.48 ^abc^	564.66 ^c^
	VITE	22.06	22.30	22.22	20.80
	GSH	181.90	181.26	188.28	196.83
	TAC	0.49 *	0.50	0.43 *	0.45
	AOPP	103.45^a^	136.05 ^ab^	124.68	115.26 ^b^
	MDA	18.96 *	20.66 *^a^	18.94	18.82 ^a^
	8OHdG	21.27	20.23	21.45	19.38
**E** **(*n* = 53)**	FOX_1_	8.81 ^ab^	7.82 ^a^	7.69 ^b^	8.22
	SOD	554.82 ^a^	520.69 ^b^	499.93 ^c^	666.69 ^abc^
	VITE	22.30	22.55	23.05	23.72
	GSH	191.38 *^ab^	211.13 *	222.13 ^a^	220.51 ^b^
	TAC	0.40 ^ab^	0.39 *	0.33 ^a^*	0.34 ^b^
	AOPP	113.84 ^a^	146.98 ^a^	132.84	133.83
	MDA	19.67	19.26	18.81	19.45
	8OHdG	16.92	19.08	18.37	16.57
**EI/II** **(*n* = 35)**	FOX_1_	8.77 *^a^	8.09 *	7.72 ^a^	8.38
	SOD	534.63	507.43 ^a^	526.34	619.89 ^a^
	VITE	21.56	22.83	21.94	23.78
	GSH	189.48 ^a^	201.78	214.18	219.52 ^a^
	TAC	0.40	0.44 ^ab^	0.35 ^a^	0.34 ^b^
	AOPP	104.96 ^ab^	147.19 ^a^	135.23	149.88 ^b^
	MDA	19.81	19.29	19.67	18.96
	8OHdG	15.81	18.53	17.06	16.05
**EIII/IV** **(*n* = 18)**	FOX_1_	9.23 ^a^	7.45 ^a^	7.89	8.36
	SOD	580.07 ^a^	537.15 ^b^	485.83 ^c^	812.78 ^abc^
	VITE	22.16	22.74	23.98	23.77
	GSH	179.26 ^ab^	216.25	235.45 ^a^	231.82 ^b^
	TAC	0.39 ^a^	0.36	0.31	0.29 ^a^
	AOPP	119.97	152.00	126.76	121.47
	MDA	18.99	16.63	16.43	18.00
	8OHdG	16.92	20.19	18.80	17.36

Note: GnRHa: gonadotropin-releasing hormone agonist. hCG: human chorionic gonadotropin. Data presented as mean and compared among groups using a linear regression mixed-effects model and an orthogonal contrast post-test. Significance was defined as *p*-value < 0.05. FOX_1_ (total hydroperoxides): μmol/g protein. MDA (malondialdehyde): nmol/g protein. AOPP (advanced oxidation protein products): μmol/L. 8OHdG (8-hydroxy-2′-deoxyguanosine): ng/mL. GSH (reduced glutathione): nmol/g protein. VitE (vitamin E): μmol/L. SOD (superoxide dismutase): U/mL. TAC (total antioxidant capacity): mEq Trolox/L. The same superscript letters in a line represent significant differences. *: *p* = 0.05.

## Data Availability

Data is contained within the article.
